# MOI is a comprehensive database collecting processed multi-omics data associated with viral infection

**DOI:** 10.1038/s41598-024-65629-6

**Published:** 2024-06-26

**Authors:** Xuefei Guo, Yang Zhao, Fuping You

**Affiliations:** Institute of Systems Biomedicine, Department of Immunology, School of Basic Medical Sciences, Beijing Key Laboratory of Tumor Systems Biology, NHC Key Laboratory of Medical Immunology, Peking University Health Science Center, Beijing, China

**Keywords:** Data integration, Databases, Computational biology and bioinformatics, Immunology

## Abstract

Viral infections pose significant public health challenges, exemplified by the global impact of COVID-19 caused by SARS-CoV-2. Understanding the intricate molecular mechanisms governing virus-host interactions is pivotal for effective intervention strategies. Despite the burgeoning multi-omics data on viral infections, a centralized database elucidating host responses to viruses remains lacking. In response, we have developed a comprehensive database named ‘MOI’ (available at http://www.fynn-guo.cn/), specifically designed to aggregate processed **M**ulti-**O**mics data related to viral **I**nfections. This meticulously curated database serves as a valuable resource for conducting detailed investigations into virus-host interactions. Leveraging high-throughput sequencing data and metadata from PubMed and Gene Expression Omnibus (GEO), MOI comprises over 3200 viral-infected samples, encompassing human and murine infections. Standardized processing pipelines ensure data integrity, including bulk RNA sequencing (RNA-seq), single-cell RNA-seq (scRNA-seq), Chromatin Immunoprecipitation sequencing (ChIP-seq), and Assay for Transposase-Accessible Chromatin using sequencing (ATAC-seq). MOI offers user-friendly interfaces presenting comprehensive cell marker tables, gene expression data, and epigenetic landscape charts. Analytical tools for DNA sequence conversion, FPKM calculation, differential gene expression, and Gene Ontology (GO)/ Kyoto Encyclopedia of Genes and Genomes (KEGG) enrichment enhance data interpretation. Additionally, MOI provides 16 visualization plots for intuitive data exploration. In summary, MOI serves as a valuable repository for researchers investigating virus-host interactions. By centralizing and facilitating access to multi-omics data, MOI aims to advance our understanding of viral pathogenesis and expedite the development of therapeutic interventions.

## Introduction

Coronavirus disease 2019 (COVID-19), caused by severe acute respiratory syndrome coronavirus 2 (SARS-CoV-2), has posed an ongoing global health threat since its emergence in late 2019, infecting over 6.3 million individuals worldwide as of July 15, 2022^1–3^. Understanding the intricate molecular mechanisms of SARS-CoV-2 infection is paramount for controlling its spread and managing severe cases. Host cells mount defensive responses upon viral invasion to combat the pathogen^4–7^. Recent advancements in next-generation sequencing technology have facilitated the exploration of host cell gene expression profiles, offering invaluable insights into virus-host interactions in vitro and in vivo^8–11^.

The GEO database is a cornerstone resource for viral-related NGS data, housing extensive gene expression and genomics datasets. From this wealth of information, specialized databases have emerged to delve into specific aspects of viral biology. VISDB focuses on viral integration sites^12^, while VirHost facilitates research on viral pathogenesis^13^. ViPR integrates genomic and proteomic data for comprehensive analysis^14^, while PhEVER delves into viral infection pathology and evolution^15^. HPIDB catalogs interactions between host proteins and pathogens^16^, while ViMIC highlights viral mutation accumulation^17^. ViralZone offers curated information on viral protein families^18^, and PHI-base specializes in pathogen-host interactions^18^. MVIP integrates multi-omics data to investigate host responses to viruses, providing insights into viral pathogenesis and host-virus interactions^19^. However, a cavity looms large in databases tailored to sorting and cataloging gene expression profiles, epigenetic landscapes, and cell markers for scRNA-seq during viral infection.

ScRNA-seq is a powerful tool for dissecting gene expression dynamics at the single-cell level, revolutionizing research in oncology, immunology, neurobiology, and developmental biology^20–23^. However, analyzing scRNA-seq data involves intricate processes such as alignment, quality control, normalization, dimensionality reduction, clustering, and cell annotation, which are crucial for drawing accurate conclusions^24–29^. While software packages like SingleR and scMatch aid in cell annotation through machine learning algorithms trained on existing scRNA-seq data^30,31^, there is a pressing need for comprehensive databases that streamline this process.

To address this gap, we introduce the MOI database (http://www.fynn-guo.cn/), an intuitive platform providing comprehensive expression profiles and abundant cell markers from multi-omics data under various virus infection conditions. MOI enables users to browse, retrieve seamlessly, and download information about viral infections, including cell markers, gene expression profiles, and epigenetic landscapes. Moreover, MOI integrates a suite of online tools for downstream multi-omics data analysis and visualization, such as FPKM calculation, detection of Differentially Expressed Genes (DEGs), Gene Ontology (GO) annotation, Kyoto Encyclopedia of Genes and Genomes (KEGG) pathway enrichment, and visualization through 16 plots. By offering these resources, MOI aims to alleviate the burdens associated with omics data analysis, ultimately facilitating more profound insights into molecular events during infection and empowering downstream applications. We anticipate that MOI will be a valuable resource for researchers engaged in virus-related multi-omics studies, fostering a deeper understanding of viral pathogenesis and developing innovative therapeutic strategies.

## Data collection and database content

### Data collection

**Figure 1:**
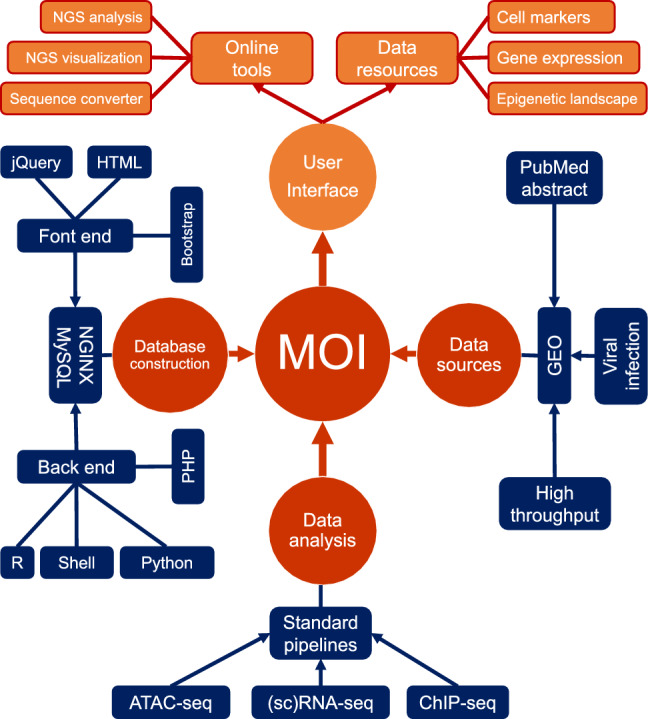
The design and structure of MOI. This schematic diagram comprehensively portrays MOI’s design and structure, elucidating its fundamental constituents and organizational schema. Data Acquisition: The raw data utilized in this study were sourced from the Gene Expression Omnibus (GEO) database, adhering to stringent inclusion criteria to ensure data quality and relevance. Data Integration: At the core of MOI lies a robust data integration pipeline that harmonizes disparate multi-omics datasets from diverse origins into a cohesive and standardized repository. Database Construction: The integrated multi-omics data are housed within a meticulously structured database architecture developed with MySQL, PHP, NGINX, Bootstrap, and so on, facilitating efficient data management, retrieval, and manipulation. User Interface: MOI boasts an intuitive user interface designed to streamline user interaction and enable seamless navigation across its functionalities. The user interface provides intuitive access to many tools and features, including data querying, analysis tools, and visualization modules.

We rigorously adhered to a series of standardized procedures to systematically collect virus-related multi-omics data, ensuring consistency and reliability throughout the data collection process^19,32^. Initially, we conducted a comprehensive search in the PubMed database, retrieving 2500 abstracts containing the keyword 'virus' as of May 2021. These candidate articles underwent meticulous filtration based on the availability of multi-omics data and the methodology utilized for library construction. Specifically, we selected articles employing high-throughput sequencing or wet experimental techniques such as bulk RNA-seq, single-cell RNA-seq (scRNA-seq), ATAC-seq, and ChIP-seq assays to extract validated information (Fig. 1).

The current database release comprises data extracted from 1757 articles related explicitly to NGS technology. Each article’s full text underwent a thorough manual review by at least two independent researchers to extract comprehensive information concerning virus infection. Each database entry includes essential metadata, including species, cell line, infection duration, library type, reference genome, PubMed ID of the publication, and details regarding the experimental or computational methodology employed for detection. This stringent methodology ensures the inclusion of high-quality, curated data within the database, facilitating robust analysis and interpretation for researchers in viral infections and multi-omics research.

### Data processing and annotation

The sequencing data generated in FASTQ file format was filtered by FastQC (https://www.bioinformatics.babraham.ac.uk/projects/fastqc/) (V0.11.9) and Trim-Galore(https://www.bioinformatics.babraham.ac.uk/projects/trim_galore/) (V0.6.4) software for quality control. The parameters of Trim-Galore are as follows: `trim_galore --gzip --trim-n --phred33 -j 7 --paired ${var}_1.fq.gz ${var}_2.fq.gz -o $wrk_dir/clean_result/`. Then, the mouse genome version mm10 was used as a reference genome to align clean data with Subread (V2.0.0) software^33^. The gene count matrix was acquired using the featureCounts (V2.0.0) program^34^. The specific arguments for Subread and featureCounts are as shown below: `subread-align -i $idx_dir -r $cle_dir/${var}_1_val_1.fq.gz -R $cle_dir/${var}_2_val_2.fq.gz -o $aln_dir/${var}.bam -T 30 -t 0`; `featureCounts -p -t exon -g gene_id -a $gtf_dir -o $cnt_dir/count_refGene $aln_dir/*.bam -T 29`. Then, the gene count data was normalized using the Fragments Per Kilobase Million (FPKM) formula^35^. R package DESeq2 (V1.38.3) was used to identify the DEGs (with the criteria: abs(log2(FoldChange)) > 2 and *p*-value < 0.05)^36^. The GO annotation and KEGG pathway enrichment analysis were conducted by the DAVID (https://david.ncifcrf.gov/summary.jsp) database (V6.8)^37–39^.

Single-cell sequencing data were aligned and quantified with Cell Ranger (v6.0.1) against the GRCh38 human and mm10 reference genome downloaded from 10× Genomics official website (https://www.10xgenomics.com/). The parameters of Cell Ranger are as follows: `cellranger count --id=$var --transcriptome = $idx_dir --fastqs=$raw_dir --sample=$var --expect-cells = 1000 --localcores 28 --localmem 88`. Preliminary counts were then used for downstream analysis. Quality control was applied to cells based on three metrics: the total UMI counts, the number of detected genes, and the proportion of mitochondrial gene count per cell. Specifically, cells with less than 1000 UMI counts and 500 detected genes and cells with more than 10% mitochondrial gene counts were filtered. To integrate cells into a shared space from different datasets for unsupervised clustering, we used the harmony algorithm (V1.2.0) to do batch effect correction^26^. We performed variable gene selection separately for each sample to detect the most variable genes used for the harmony algorithm. Then, we use the R package Seurat (V5.0.3) to conduct the normalization, dimensional reduction clustering, etc. (https://satijalab.org/seurat/index.html)^25^. The annotation of the prepared single cell was completed by one shared R package named SingleR (V2.0.0) (https://github.com/dviraran/SingleR)^29^. The DEGs (with the criteria: abs(log2(FoldChange)) > 0.5 and *p*-value < 0.05) of scRNA-seq were detected by MAST (V1.24.1) (https://github.com/RGLab/MAST)^40^.

As for the ChIP-seq and ATAC-seq data downloaded from the available database, we first conducted the quality control analysis for the raw data in fastq format with Trim-Galore (V0.6.4). Then, the filtered reads were aligned and quantified to their corresponding genome, such as hg38 or mm10, using aligner bowtie2 (V2.3.5.1)^41,42^, which would output the SAM or BAM files. The parameters of bowtie2 are as follows: `bowtie2 --very-sensitive -X 2000 -x $Bowtie2Index -1 $cln_res/${sample}_1_val_1.fq.gz -2 $cln_res/${sample}_2_val_2.fq.gz -p $PPN | samtools view -buSh -@ $PPN | samtools sort -@ $PPN -O BAM -o $aln_res/${sample}.sorted.bam`. Then, the samtools (V1.10) were used to sort and index the SAM/BAM files with the following parameter: `samtools index -@ $PPN $aln_res/${sample}.sorted.bam`^43^. Peak calling was completed with MACS3 (V3.0.0a5)^44^ with the default parameters, which produced the peak files with bed format for each sample. In the downstream of ATAC-seq and ChIP-seq, we adopted different strategies. The ATAC-seq data were first merged by the bedtools (V2.31.1) merge tool for all samples^45^. Then, they were normalized by the bamCoverage (V3.3.2) tool in deepTools (V3.3.2) suites^46^, using the parameter `bamCoverage --bam $var -o ${var%.*}.bw --binSize 100 --normalizeUsing RPKM --effectiveGenomeSize 2864785220 --ignoreForNormalization chrM –extendReads`. Then, the normalized data could be used for differential analysis with DESeq2 (V1.38.3)^36^. For ChIP-seq data: The reads that fell in the peak gap were normalized by RPKM first using bamCoverage (V3.3.2) with the above parameters. The treatment samples were compared to their Input data using the bamCompare (V3.3.2) tool with the parameter: `bamCompare -b1 idx-MUTMSCLACH.bam -b2 idx-MUTMSCLAIN.bam -o bwResIpInput/MUTIPratioInput --binSize 145 --normalizeUsing RPKM --effectiveGenomeSize 2864785220 --ignoreForNormalization chrM --extendReads -p 22 --scaleFactorsMethod None`. Then, the R package ChIPseeker (V1.34.1) was used to annotate the identified peak files^47^. Last, the Integrative Genomics Viewer (IGV) (V2.17.4) was used to visualize these peaks from ChIP-seq or ATAC-seq^48^.

### Database statistics

**Figure 2:**
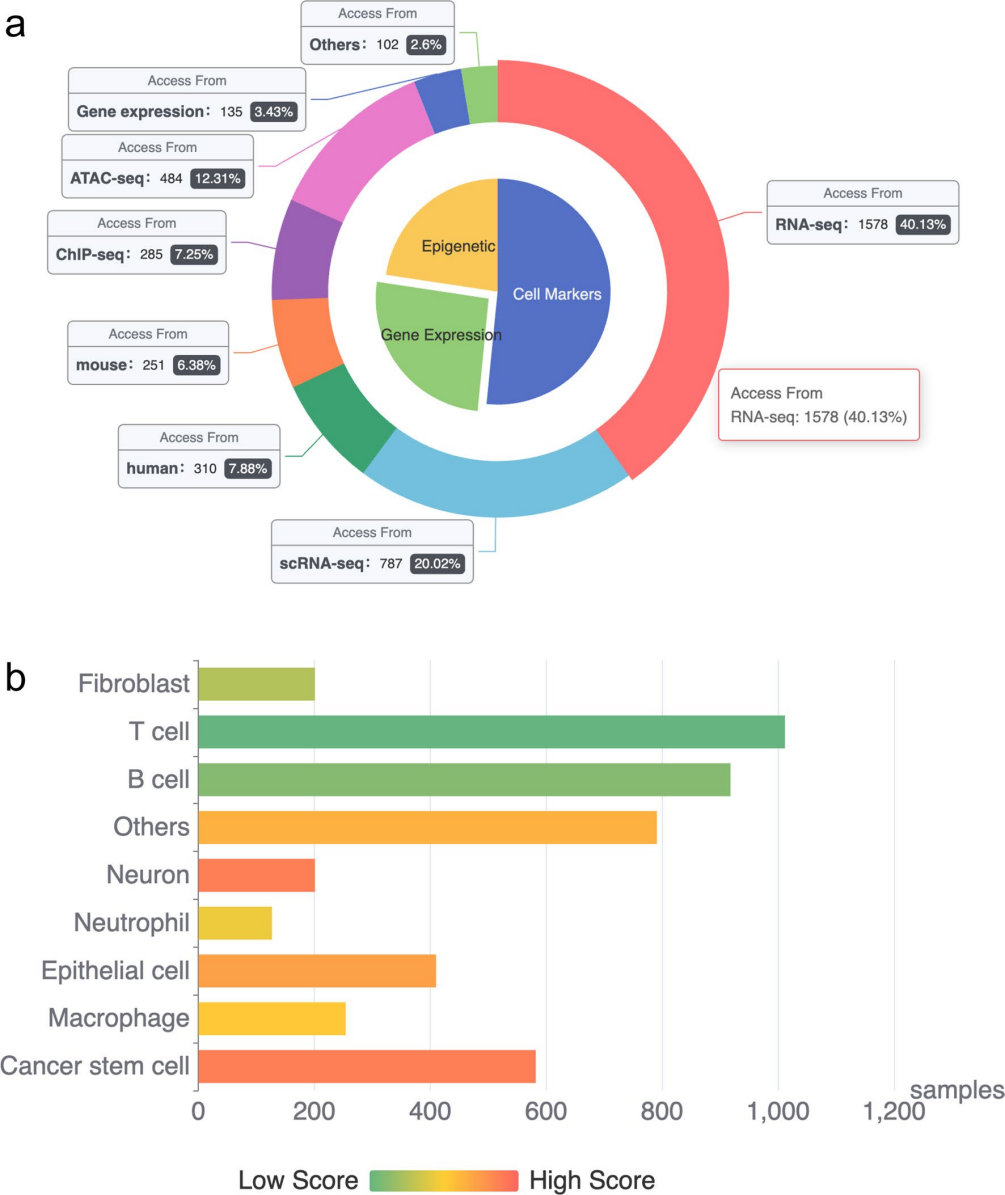
The composition and statistics of MOI. (**a**) Comprehensive statistics of multi-omics samples, which delineates detailed statistics about the multi-omics samples employed within the MOI study. The encompassed methodologies include bulk RNA-seq, single-cell RNA-seq (scRNA-seq), Chromatin Immunoprecipitation sequencing (ChIP-seq), and Assay for Transposase-Accessible Chromatin using sequencing (ATAC-seq). (**b**) The total cell markers enumeration across immune cell types.

The data sources of the MOI database primarily originate from original data from the GEO database and published literature. Following retrieval, the data are centralized onto a local server where a standardized analysis and annotation process is employed. Presently, the database comprises 1578 bulk RNA-seq samples, 787 single-cell RNA-seq (scRNA-seq) samples, 285 ChIP-seq samples, and 484 ATAC-seq samples (Fig. 2a).

The compiled data are predominantly categorized into three main classes: (1) specific cell markers of mouse and human cells across various tissues and experimental treatments; (2) gene expression profiles of cells post-viral infection at diverse time points; and (3) dynamics of cell transcription factors and epigenetic landscapes of human and mouse cells. As of December 2022, MOI encompasses 1597 human cell marker records, 897 mouse cell marker records, and 524 cell marker annotations derived from single-cell sequencing (Fig. 2b). The database includes 1638 human and 1543 mouse transcription factors (Supplementary Fig. 1b and 1d). Furthermore, MOI incorporates a comprehensive array of innate immune cells, commonly utilized laboratory cell lines, and gene expression profiles spanning diverse time points and viral infections (Supplementary Fig. 1a and 1c). Moving forward, the database is poised for continuous expansion and enrichment. The repository will be augmented with additional scRNA-seq, ChIP-seq, and ATAC-seq data to supplement the growing repertoire of cell types and treatment time points.

## Database features and applications

### User-friendly browsing and searching

**Figure 3:**
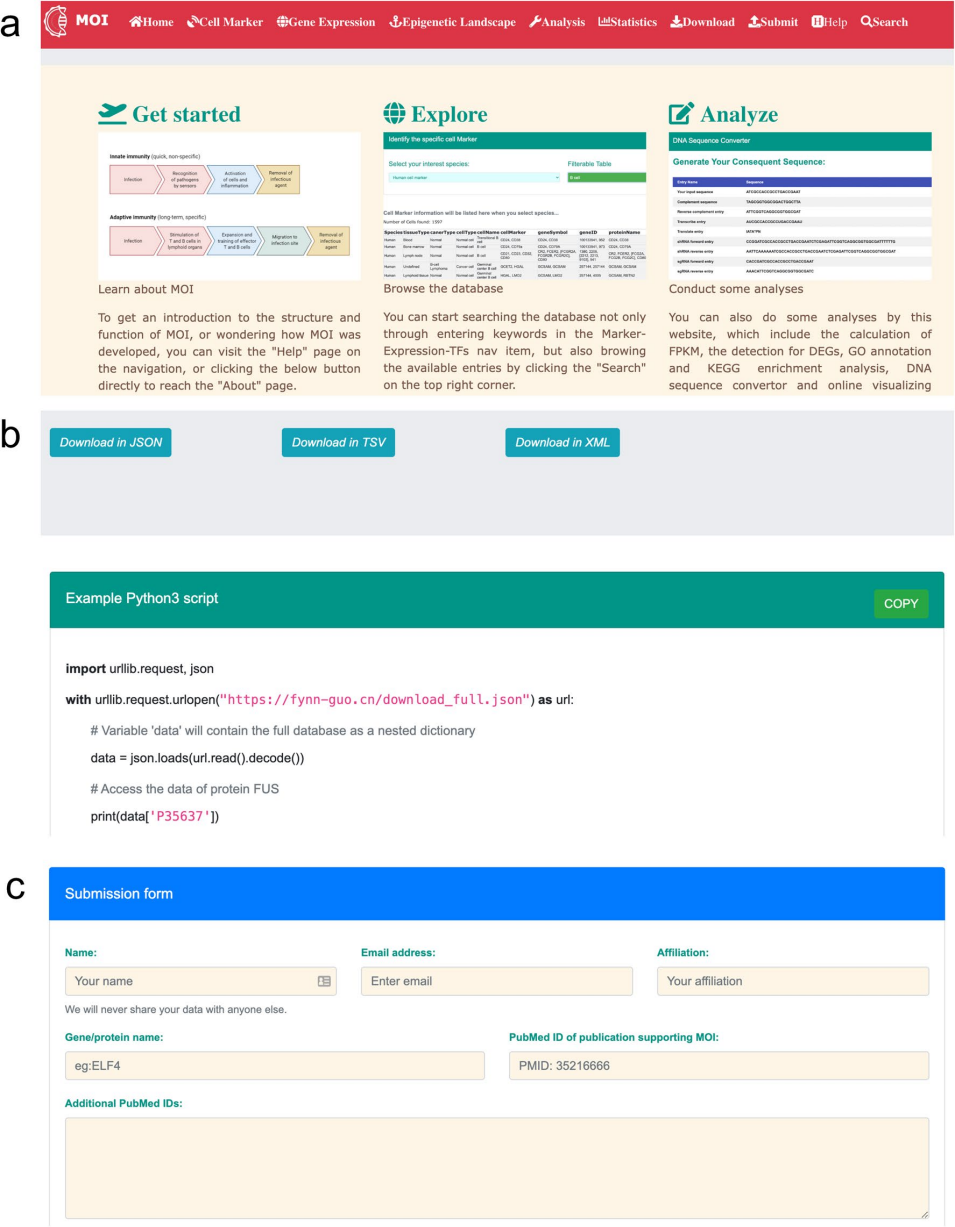
The user interface and navigation of MOI. (**a**) The home page interface of MOI. This section illustrates the interface of the MOI home page, providing an overview of its layout and features. (**b**) The data download options of MOI. Various methods for downloading data from MOI are showcased, offering users convenient access to integrated multi-omics datasets. (**c**) The data submission form of MOI. A form enabling users to submit their data to MOI is displayed, facilitating the expansion and enrichment of the integrated database.

An intelligible web interface has been meticulously crafted to facilitate scholarly inquiry into information pertinent to viral infection. Access to this comprehensive repository is afforded through a freely accessible database website, accessible at http://www.fynn-guo.cn/home.php. The website’s interface is structured into ten distinct sections, comprising ‘Home’, ‘Cell Marker’, ‘Gene Expression’, ‘Epigenetic Landscape’, ‘Analysis’, ‘Statistics’, ‘Download’, ‘Submit’, ‘Help’, and ‘Search’ (Fig. 3a).

The Home page serves as the principal gateway, providing users with fundamental insights into the MOI database and a concise overview of its contents. Upon further navigation, users are directed to sub-sections such as ‘Get Started’, ‘Explore’, and ‘Analyze’, each seamlessly linked to corresponding pages, namely ‘Help’, ‘Markers’, and ‘Analysis’. This structured approach facilitates efficient navigation and access to relevant information. Furthermore, interactive visual aids are seamlessly integrated to elucidate the features and functionalities of MOI, thereby enhancing user engagement and comprehension. Notably, a dynamic world map located at the bottom of the Home page meticulously logs the access history of MOI, offering valuable insights into user interactions and usage patterns.

A user-friendly graphical navigation system has been implemented to expedite the exploration of MOI's extensive data, empowering users to navigate to their specific areas of interest effortlessly. For example, the Marker page facilitates the exploration of cell markers associated with a diverse array of cells derived from human or mouse tissue under various conditions, meticulously categorized by species. Additionally, users can utilize the ‘Filterable Table’ feature to input keywords, such as ‘T cell’ or ‘CD14’, thereby streamlining the process of retrieving relevant information tailored to their research needs. This functionality enhances the efficiency and precision of data exploration within the MOI database.

Similarly, the Expression and Transcription Factors (TFs) pages replicate the functionality observed in the Marker page, allowing users to explore gene expression profiles and glean insights into transcription factor activity. This consistent functionality ensures coherence and uniformity in the user experience across distinct sections of the MOI database. Moreover, users can expedite their search endeavors by leveraging the 'Search' button at the interface’s apex. Upon activation, this feature directs users to the search page, where they can execute finely tailored queries within MOI’s extensive database. This refined approach enhances the efficiency and efficacy of information retrieval, empowering users to access pertinent data to their research inquiries swiftly.

### Data download and submission

On the one hand, users are directed to the Download page to access specific information within the MOI database. Here, data is systematically categorized based on species, tissue, cell line, and identification method. Datasets are available in various standard-compliant formats, including JSON, TSV, and XML (Fig. 3b). To ensure the integrity of downloaded files, users are provided with corresponding MD5 checksum files for verification purposes. Users can also directly access specific MOI data through Python packages such as urllib and JSON.

On the other hand, the Submit page facilitates user contributions of new virus-related data by soliciting the submission of the PubMed ID, accessible data URL, and contributor information (Fig. 3c). This process undergoes a standardized internal collection and verification procedure. Subsequently, additional data is curated and slated for publication in the subsequent stable release within a maximum timeframe of 10 months. Simultaneously, MOI offers a ‘Help’ page containing documentation detailing the platform’s user guide information to enhance user experience and usability. Users are encouraged to engage with the platform, explore its features, and provide feedback for ongoing improvements.

### Online data analysis and visualization tools

**Figure 4:**
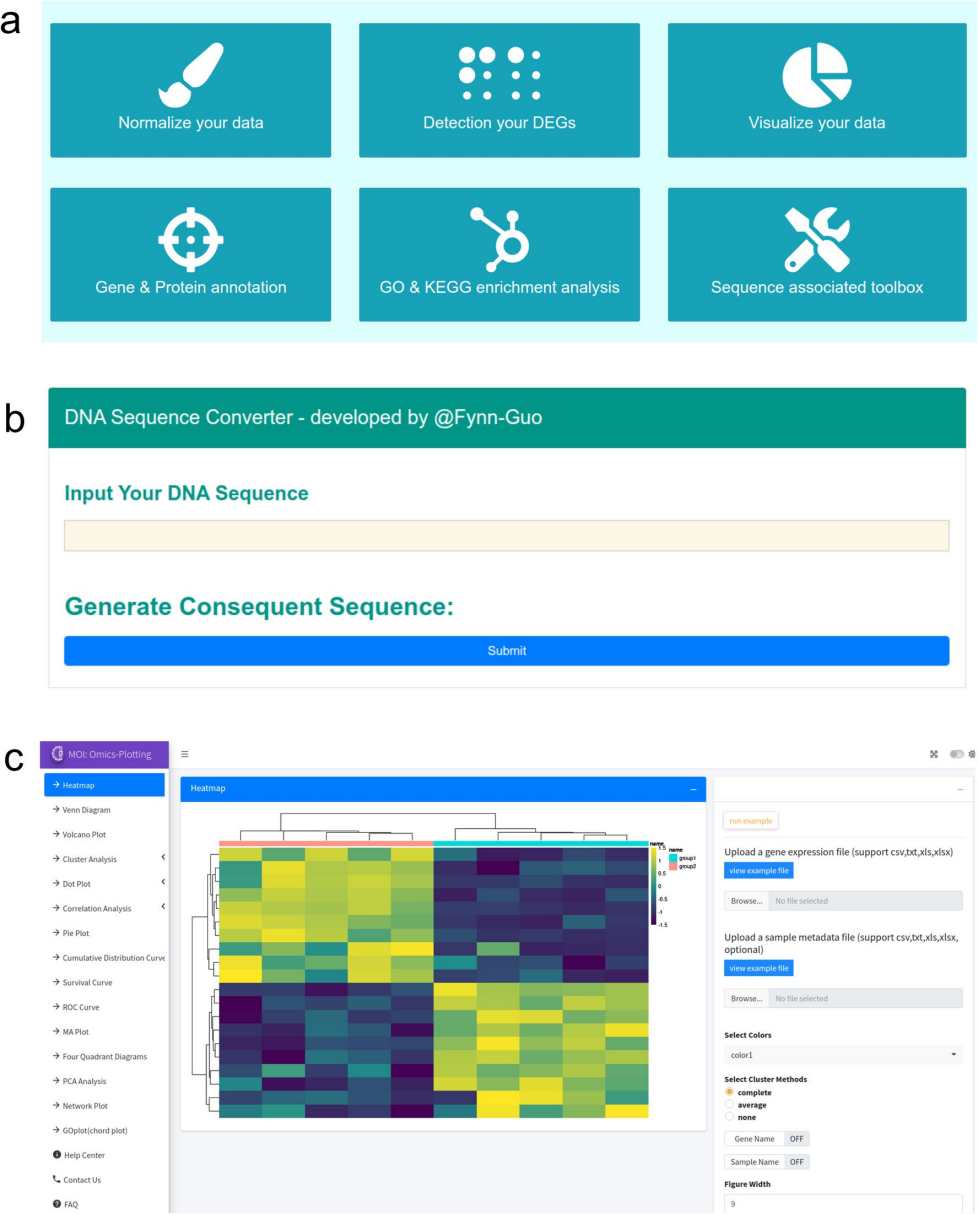
The online analysis and visualization tools of MOI. (**a**) An overview of downstream analysis and visualization tools. (**b**) An interface of a DNA sequence converter that could convert DNA sequences into various formats. (**c**) The interface of Omics-Plotting. An overview of the Omics-Plotting tool is presented, showcasing its capability to generate 16 standard plots relevant to multi-omics analysis, enabling effective visualization and interpretation of complex data sets.

In addition to the aforementioned data sources, MOI presents a suite of online data analysis tools (Fig. 4a), primarily tailored for downstream analysis of bulk RNA-seq data. These tools encompass a range of functionalities, including the computation of FPKM values within expression matrices containing biological replicates. Users can leverage these tools for tasks such as detecting DEGs, elucidating validated gene functions, and conducting GO and KEGG annotations on DEGs. Notably, the gene list length for annotation should be at most 2000 characters.

Since its inception, molecular biology has emerged as a cornerstone of fundamental research in life sciences. Primer design has thus become an indispensable skill for researchers engaged in wet laboratory experiments. Consequently, MOI offers a user-friendly DNA sequence processing tool to aid researchers in primer sequence design. This tool facilitates various tasks, including identifying reverse complementary, transcription, translation, and shRNA or sgRNA sequences designed based on plasmid characteristics and restriction endonuclease sites (Fig. 4b).

Furthermore, MOI has developed a data visualization toolbox to provide users with customizable online representations of 16 typical scientific graphs and charts. These visualizations encompass volcano plots, heatmaps, bubble plots, Venn diagrams, boxplots, violin plots, chord diagrams, and more (Fig. 4c). By offering these comprehensive and freely accessible online data analysis and visualization tools, MOI empowers researchers lacking bioinformatic expertise and programming skills to conduct complex analyses and generate publication-ready plots independently and conveniently.

## System design and implementation

As shown in Fig. 1, the MOI website is hosted on a Linux-based NGINX (V1.21.0) web server, accessed at https://www.nginx.com/. Its user interface is meticulously designed with optimization techniques utilizing the Bootstrap (V4.6.1) framework, accessible at https://getbootstrap.com/docs/4.6/getting-started/introduction/. To facilitate sophisticated data presentation, we have integrated plugins for prominent JavaScript libraries, such as jQuery (V3.6.1), incorporating DataTables (V1.10.19) (https://datatables.net), Biodalliance (v0.13.8) (http://www.biodalliance.org/about.html), and morris.js (v0.5.0) (https://morrisjs.github.io/morris.js/index.html). In the server-side architecture, we deploy PHP (V7.4.24) (http://www.php.net), Python (V3.9.7) (https://www.python.org/), and R (V4.1.3) (https://www.r-project.org/). The MOI database is securely housed within a MySQL (V8.0.24) (http://www.mysql.com) database infrastructure. A meticulous testing regime has been conducted across diverse mainstream web browsers, including Google Chrome, Firefox, Opera, Apple Safari, and Microsoft Edge to ascertain optimal performance and cross-browser compatibility. Access to the MOI database is readily extended to the scholarly community through the online portal at http://www.fynn-guo.cn/. This repository is a pivotal resource for conducting molecular immunology investigations focusing on viral infections.

## Case study: exploring host responses to viral infections utilizing MOI

*Introduction* Viral infections present substantial global health challenges, epitomized by the widespread ramifications of diseases like COVID-19 stemming from SARS-CoV-2. Comprehending the intricate molecular interplay between viruses and their hosts is a cornerstone for devising efficacious intervention strategies. Despite the proliferation of multi-omics data pertinent to viral infections, a conspicuous absence of a centralized repository still facilitates exhaustive exploration into host responses. We introduce the MOI database to address this lacuna. MOI is conceived to furnish researchers with a meticulously curated compendium of multi-omics data delineating virus-host interactions.

*Accessing MOI* Researchers gain entry to MOI via the web interface accessible at http://www.fynn-guo.cn/. Upon ingress, users are greeted with an intuitive, user-friendly interface meticulously crafted to expedite seamless navigation and data retrieval.

*Dataset overview* MOI encompasses a vast repository of over 3200 samples afflicted with viral infections sourced from human and mouse models. These samples undergo scrupulous curation and are collated from eminent repositories such as PubMed and the GEO. Spanning an array of high-throughput sequencing modalities, including (single-cell) RNA-seq, ChIP-seq, and ATAC-seq, MOI offers a comprehensive panorama of multi-omics data pertinent to viral infections.

*Data processing and integrity* Upholding data integrity, MOI implements standardized processing pipelines tailored for data normalization and stringent quality control measures. This rigorous protocol ensures the reliability and suitability of the database contents for downstream analytical endeavors.

*Features and tools* MOI boasts an array of analytical tools and features meticulously designed to facilitate profound exploration and interpretation of multi-omics data. These encompass:

- Cell Marker Tables: Exhaustive tabulations delineating cell markers attributed to diverse cell types implicated in viral infections.
- Gene Expression Data: Access to gene expression profiles spanning various viral infections allows users to scrutinize gene expression signature alterations.
- Epigenetic Landscape Charts: These are visual representations of epigenetic landscapes. They elucidate chromatin accessibility and transcription factor binding motifs, unraveling intricate regulatory mechanisms.
- Analytical Tools: A repertoire encompassing DNA sequence conversion utilities, FPKM calculation modules, differential gene expression analysis platforms, and enrichment analysis tools for elucidating GO/KEGG pathways.
- Visualization Plots: Sixteen bespoke visualization plots, meticulously crafted to facilitate intuitive exploration and comprehension of multi-omics data landscapes.

## Discussion

Investigating viral infections is fundamental to biological inquiry and is pivotal for understanding disease mechanisms. However, until recently, comprehensive elucidation of the complex interplay among gene expression, cell markers, and regulatory elements has posed significant challenges. The emergence of state-of-the-art experimental techniques and computational methodologies has heralded a transformative era, shedding light on the intricate dynamics of viral-host interactions. Recent advancements in NGS research have propelled numerous datasets to the forefront, offering unparalleled insights into the molecular terrain of viral infections^1–5^.

Furthermore, the landscape of databases and web portals dedicated to viruses, viral infections, and host organisms is rich and diverse, each offering unique features and focusing on distinct research areas. For instance, VISDB is a dedicated repository for curating information on viral integration sites within the human genome^12^. In contrast, VirHost is tailored to facilitate research on viral pathogenesis by providing comprehensive insights into host-virus interactions, including identifying host factors implicated in viral infection and targeting host factors by viral proteins^13^. Similarly, ViPR stands out as a comprehensive database that consolidates genomic, proteomic, and functional data spanning various viruses. Equipped with data analysis and visualization tools, ViPR empowers researchers to delve into multiple aspects of viral evolution, epidemiology, and pathogenesis^14^. In a parallel vein, PhEVER fills a niche by focusing on phylogenetic analysis of viral evolution and its ramifications for disease dynamics. Through its suite of tools for sequence analysis and evolutionary prediction, PhEVER enables researchers to discern the evolutionary trajectories of viral sequences^15^. Likewise, HPIDB serves as a repository cataloging human protein interactions, including those pivotal in viral infections. By furnishing information on protein–protein interactions relevant to viral pathogenesis and host response, HPIDB aids in unraveling the intricate interplay between viruses and their hosts^16^. Additionally, resources like ViMIC cater to viral microbiology, providing comprehensive insights into viral genomes, proteins, and interactions^17^. Similarly, ViralZone is a meticulously curated database offering exhaustive information on viral protein families and their functions. This resource is indispensable for researchers seeking to elucidate the molecular mechanisms underpinning viral infections^18^. Moreover, PHI-base is a valuable resource by focusing on interactions between pathogens and their hosts, including viruses. By providing curated information on genes implicated in pathogen-host interactions, PHI-base facilitates the study of host defense mechanisms and pathogen virulence factors^49^. On a broader scale, MVIP represents a pioneering initiative to integrate multi-omics data to unravel the intricate landscape of host responses to viral infections. By amalgamating RNA-seq, ChIP-seq, and CLIP-seq data, MVIP endeavors to shed light on the molecular mechanisms underlying viral infections and host-virus interactions^19^. However, despite the resources available, a notable gap persists in databases explicitly dedicated to organizing and cataloging gene expression profiles, epigenetic landscapes, and cell markers for single-cell RNA sequencing (scRNA-seq) during viral infections.

In response to this notable void, we have developed what we believe to be the first comprehensive database dedicated to the intricacies of gene expression, epigenetic landscape, cell markers, and transcription factors. Through meticulously crafted systematic data collection protocols and standardized datasets, alongside designing a user-friendly web platform adhering to rigorous research standards, our aspiration for MOI is to serve as a cornerstone resource enriching the endeavors of biologists and data scientists alike. MOI is envisioned as a tool to foster a deeper comprehension of the intricate interplay between viruses and their hosts, elucidating gene expression alterations and the regulatory mechanisms underlying these interactions. Additionally, we aim to provide researchers with the means to construct more exhaustive gene regulatory networks by amalgamating the wealth of data accessible through MOI.

Illustrating the breadth of MOI's scope, the database boasts an extensive repository encompassing thousands of cell marker records for human and mouse cells, along with comprehensive catalogs comprising 1638 human transcription factors and 1543 mouse transcription factors. Moreover, MOI includes diverse innate immune cells and standard laboratory cell lines, facilitating the exploration of gene expression profiles across different treatment time points and in response to various viral strains. To further enhance utility, MOI seamlessly integrates a suite of bioinformatic analysis and visualization tools within its framework, offering researchers resources to dissect and interpret the wealth of data.

Following the inaugural release of MOI, our immediate focus is on enhancing its capabilities to encompass more comprehensive epigenomic annotations. This includes delving into diverse facets such as transcription factor binding, histone modification, and chromatin accessibility across various cell lines in response to viral infections^50^. One notable area of emphasis is integrating information about transposable elements (TEs) and endogenous retroviruses (ERVs) into MOI^51–53^. Emerging research suggests that TEs undergo up-regulation shortly after viral infection, preceding significant increases in virus replication and interferon β expression^54^. This underscores their potential role in modulating host innate immune responses, a phenomenon we aim to elucidate within MOI's curated datasets. By incorporating detailed information on TEs and ERVs, we strive to provide researchers with a nuanced understanding of their contributions to viral pathogenesis. Furthermore, we remain committed to refining MOI’s user experience by enhancing visualization tools for scRNA-seq gene expression comparisons and optimizing search query speeds. These improvements aim to bolster MOI’s utility as a comprehensive data resource, catering to the needs of both traditional molecular biology research and burgeoning computational methodologies.

In summary, MOI is a testament to our dedication to advancing scientific inquiry. By continuously evolving and expanding its capabilities, we endeavor to empower researchers with the tools and resources needed to unravel the complexities of virus-host interactions, driving forward both traditional molecular biology research and the burgeoning field of computational approaches.

### Supplementary Information

Supplementary Figure 1.

## Data Availability

Users can access any feature available in the MOI database without registering or logging in. All data is freely accessible to the research community at http://www.fynn-guo.cn/json_data.json or http://www.fynn-guo.cn/download_infor.tsv. In addition to various options for downloading data on the *Download* page, users can customize and download the filtered data on the *Search* page.

## References

[1] Zhao Y (2021). SARS-CoV-2 spike protein interacts with and activates TLR41. Cell Res..

[2] Su Y (2020). Multi-omics resolves a sharp disease-state shift between mild and moderate COVID-19. Cell.

[3] Wang D (2021). The SARS-CoV-2 subgenome landscape and its novel regulatory features. Mol. Cell.

[4] Andres-Terre M (2015). Integrated, multi-cohort analysis identifies conserved transcriptional signatures across multiple respiratory viruses. Immunity.

[5] Wu A (2021). One year of SARS-CoV-2 evolution. Cell Host Microbe.

[6] Zou L (2020). SARS-CoV-2 viral load in upper respiratory specimens of infected patients. New Engl. J. Med..

[7] Ruff WE, Greiling TM, Kriegel MA (2020). Host–microbiota interactions in immune-mediated diseases. Nat. Rev. Microbiol..

[8] Mahalingam S (2021). Landscape of humoral immune responses against SARS-CoV-2 in patients with COVID-19 disease and the value of antibody testing. Heliyon.

[9] Watanabe T, Watanabe S, Kawaoka Y (2010). Cellular networks involved in the influenza virus life cycle. Cell Host Microbe.

[10] Lee S (2021). The SARS-CoV-2 RNA interactome. Mol. Cell.

[11] Stukalov A (2021). Multilevel proteomics reveals host perturbations by SARS-CoV-2 and SARS-CoV. Nature.

[12] Tang D (2020). VISDB: A manually curated database of viral integration sites in the human genome. Nucleic Acids Res..

[13] Mihara T (2016). Linking virus genomes with host taxonomy. Viruses.

[14] Zhang, Y., Zmasek, C., Sun, G., Larsen, C. N. & Scheuermann, R. H. Hepatitis C virus database and bioinformatics analysis tools in the virus pathogen resource (ViPR). In *Methods in Molecular Biology, vol. 1911* 47–69 (Humana Press Inc., 2019). 10.1007/978-1-4939-8976-8_3 30593617

[15] Palmeira L, Penel S, Lotteau V, Rabourdin-Combe C, Gautier C (2011). PhEVER: A database for the global exploration of virus-host evolutionary relationships. Nucleic Acids Res..

[16] Ammari MG, Gresham CR, McCarthy FM, Nanduri B (2016). HPIDB 2.0: A curated database for host-pathogen interactions. Database Oxf..

[17] Wang Y (2022). ViMIC: A database of human disease-related virus mutations, integration sites and cis-effects. Nucleic Acids Res..

[18] Hulo C (2011). ViralZone: A knowledge resource to understand virus diversity. Nucleic Acids Res..

[19] Tang Z (2022). MVIP: Multi-omics portal of viral infection. Nucleic Acids Res..

[20] Katzenelenbogen Y (2020). Coupled scRNA-Seq and intracellular protein activity reveal an immunosuppressive role of TREM2 in cancer. Cell.

[21] Papalexi E, Satija R (2018). Single-cell RNA sequencing to explore immune cell heterogeneity. Nat. Rev. Immunol..

[22] Li Q (2019). Developmental heterogeneity of microglia and brain myeloid cells revealed by deep single-cell RNA sequencing. Neuron.

[23] Wang M (2018). Single-cell RNA sequencing analysis reveals sequential cell fate transition during human spermatogenesis. Cell Stem Cell.

[24] Trapnell C (2014). The dynamics and regulators of cell fate decisions are revealed by pseudotemporal ordering of single cells. Nat. Biotechnol..

[25] Satija R, Farrell JA, Gennert D, Schier AF, Regev A (2015). Spatial reconstruction of single-cell gene expression data. Nat. Biotechnol..

[26] Korsunsky I (2019). Fast, sensitive and accurate integration of single-cell data with Harmony. Nat. Methods.

[27] Butler A, Hoffman P, Smibert P, Papalexi E, Satija R (2018). Integrating single-cell transcriptomic data across different conditions, technologies, and species. Nat. Biotechnol..

[28] Clarke ZA (2021). Tutorial: Guidelines for annotating single-cell transcriptomic maps using automated and manual methods. Nat. Protocols.

[29] Kharchenko PV (2021). The triumphs and limitations of computational methods for scRNA-seq. Nat. Methods.

[30] Aran D (2019). Reference-based analysis of lung single-cell sequencing reveals a transitional profibrotic macrophage. Nat. Immunol..

[31] Hou R, Denisenko E, Forrest ARR (2019). ScMatch: A single-cell gene expression profile annotation tool using reference datasets. Bioinformatics.

[32] Hu H (2019). AnimalTFDB 3.0: A comprehensive resource for annotation and prediction of animal transcription factors. Nucleic Acids Res..

[33] Liao Y, Smyth GK, Shi W (2013). The Subread aligner: Fast, accurate and scalable read mapping by seed-and-vote. Nucleic Acids Res..

[34] Liao Y, Smyth GK, Shi W (2014). FeatureCounts: An efficient general purpose program for assigning sequence reads to genomic features. Bioinformatics.

[35] Zhao Y (2021). TPM, FPKM, or normalized counts? A comparative study of quantification measures for the analysis of RNA-seq data from the NCI patient-derived models repository. J. Transl. Med..

[36] Love MI, Huber W, Anders S (2014). Moderated estimation of fold change and dispersion for RNA-seq data with DESeq2. Genome Biol..

[37] Dennis, G. *et al. DAVID: Database for Annotation, Visualization, and Integrated Discovery*. *Genome Biology, vol. 4* http://dot.ped.med.umich.edu:2000/ (2003). PMC193660 12734009

[38] Kanehisa, M. & Goto, S. *KEGG: Kyoto Encyclopedia of Genes and Genomes*. *Nucleic Acids Research, vol. 28* http://www.genome.ad.jp/kegg/ (2000). 10.1093/nar/28.1.27 PMC102409 10592173

[39] Kanehisa M (2019). Toward understanding the origin and evolution of cellular organisms. Protein Sci..

[40] Finak G (2015). MAST: A flexible statistical framework for assessing transcriptional changes and characterizing heterogeneity in single-cell RNA sequencing data. Genome Biol..

[41] Langmead B, Trapnell C, Pop M, Salzberg SL (2009). Ultrafast and memory-efficient alignment of short DNA sequences to the human genome. Genome Biol..

[42] Dobin A (2013). STAR: Ultrafast universal RNA-seq aligner. Bioinformatics.

[43] Li H (2009). The sequence alignment/map format and SAMtools. Bioinformatics.

[44] Zhang Y (2008). Model-based analysis of ChIP-Seq (MACS). Genome Biol..

[45] Quinlan AR, Hall IM (2010). BEDTools: A flexible suite of utilities for comparing genomic features. Bioinformatics.

[46] Ramírez F, Dündar F, Diehl S, Grüning BA, Manke T (2014). DeepTools: A flexible platform for exploring deep-sequencing data. Nucleic Acids Res..

[47] Yu G, Wang LG, He QY (2015). ChIP seeker: An R/Bioconductor package for ChIP peak annotation, comparison and visualization. Bioinformatics.

[48] Robinson JT (2011). Integrative genomics viewer. Nat. Biotechnol..

[49] Urban M (2020). PHI-base: The pathogen-host interactions database. Nucleic Acids Res..

[50] Scott-Browne JP (2016). Dynamic changes in chromatin accessibility occur in CD8+ T cells responding to viral infection. Immunity.

[51] Macchietto MG, Langlois RA, Shen SS (2020). Virus-induced transposable element expression up-regulation in human and mouse host cells. Life Sci. Alliance.

[52] Guo X, Zhao Y, You F (2024). Identification and characterization of endogenous retroviruses upon SARS-CoV-2 infection. Front. Immunol..

[53] Cañadas I (2018). Tumor innate immunity primed by specific interferon-stimulated endogenous retroviruses. Nat. Med..

[54] Chuong EB, Elde NC, Feschotte C (2016). Regulatory evolution of innate immunity through co-option of endogenous retroviruses. Science.

