# Supplementary material for: MOI is a comprehensive database collecting processed multi-omics data associated with viral infection
Source: Sci Rep. 2024 Jun 26;14:14725. doi: 10.1038/s41598-024-65629-6 (PMC11208532; doi:10.1038/s41598-024-65629-6)

**Supplementary figure 1. The detailed statistics of gene expression profiles and transcription factors in MOI**. a) The distribution of gene expression profiles within primary cells obtained from single-cell RNA-sequencing (scRNA-seq). b) Distribution analyses of transcription factors are presented separately for mice. c) The distribution of gene expression profiles within typical cell lines. d) Distribution analyses of transcription factors are depicted separately for the human species.

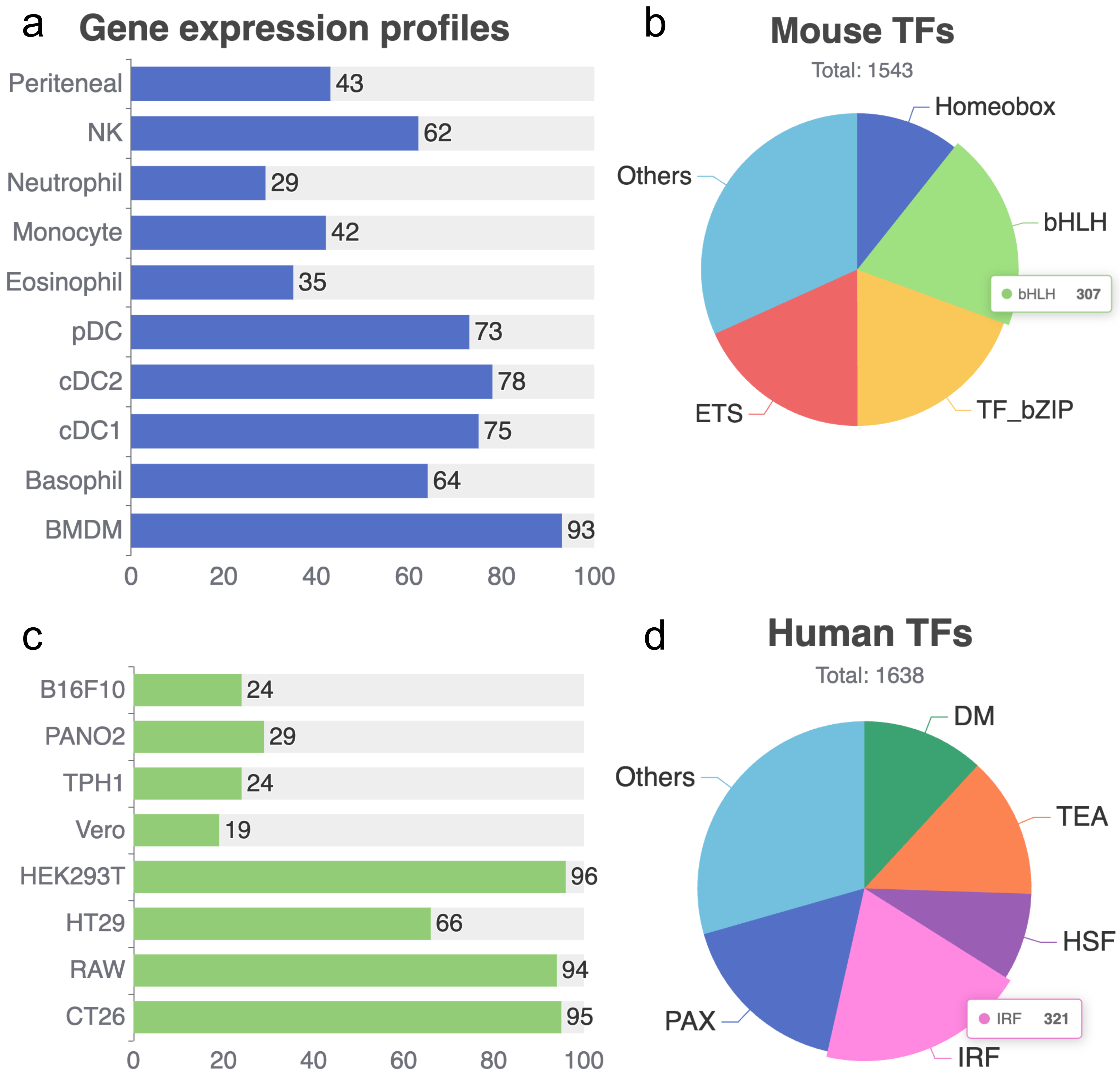

Supplement: Supplementary file 1 — Supplementary Figure 1. [file 41598_2024_65629_MOESM1_ESM.pdf]
